# Competitive Cooperation: Institutional and Social Dimensions of Collaboration in the Alliance of Science Organisations in Germany

**DOI:** 10.1007/s00048-022-00322-1

**Published:** 2022-02-10

**Authors:** Vanessa Osganian

**Affiliations:** Deutsches Museum von Meisterwerken der Naturwissenschaft und Technik, Museumsinsel 1, 80538 München, Germany

**Keywords:** Alliance of Science Organisations, Cooperation, Research Policy, Institutionalization, Federal Republic of Germany, Corporatism, Allianz der Wissenschaftsorganisationen, Kooperation, Forschungspolitik, Institutionalisierung, Bundesrepublik Deutschland, Korporatismus

## Abstract

This paper examines the institutional and social dimensions of cooperation in the Alliance of Science Organisations, the central corporatist stakeholder in German science policy, in the 1970s and 1980s, which were a crucial period for this committee. In doing so, this essay mainly focuses on the way science organizations interact with each other, as well as with national politics. The Federal Ministry of Research invited the Alliance to regular meetings and thereby fostered its involvement into political decision-making processes. Consequently, the question of who belonged to the Alliance came into the focus of different players. Although the members of the Alliance themselves decided on the composition of their committee, they were not able to completely insulate themselves from external demands. Including new members into the Alliance had a destabilizing effect on the carefully balanced distribution of power within this committee, as will be shown through the case study on the admission of the Association of the Major Research Centers (*Arbeitsgemeinschaft der Großforschungseinrichtungen*, AGF) in 1976. In order to restabilize the situation, the members of the Alliance tried to exclude the AGF from certain issues. At the same time, the AGF itself was keen on being regarded as an equal partner and thus strove for its inclusion. This complex interplay of cooperative and competitive actions finally resulted in the institutionalization of the Alliance.

## Abstract (de)

Der vorliegende Beitrag untersucht die institutionelle und soziale Dimension der Zusammenarbeit in der Allianz der Wissenschaftsorganisationen, dem zentralen korporatistischen Akteur in der bundesdeutschen Wissenschaftspolitik, in den 1970er und 1980er Jahren, einer wichtigen Scharnierphase für dieses Gremium. Dabei liegt der Fokus insbesondere auf der Analyse der vielfältigen Interaktionen zwischen den Wissenschaftsorganisationen, ebenso wie zwischen Wissenschaft und Politik.

Um seine forschungspolitische Agenda mit den Wissenschaftsorganisationen abzustimmen, etablierte das Bundesforschungsministerium regelmäßige Konsultationen mit den Präsidenten der Allianz, die eine verstärkte Einbindung des Gremiums in politische Entscheidungsfindungsprozesse zur Folge hatte. Folglich rückte auch die Zusammensetzung der Allianz immer wieder in den Fokus verschiedener Akteure. Obwohl die Allianz prinzipiell selbst über ihre Zusammensetzung entschied, konnte sie sich nicht gänzlich gegenüber von außen an sie herangetragenen Forderungen verschließen, was zu Öffnungen des Gremiums führte – wie im Falle der Aufnahme der Arbeitsgemeinschaft der Großforschungseinrichtungen (AGF) 1976. Das wiederum wirkte destabilisierend auf das zuvor sorgfältig austarierte Machtverhältnis in der Allianz. Um die Situation zu restabilisieren, initiierten die Allianzmitglieder von innen heraus gegenüber der AGF in bestimmten Fragen partielle Schließungen, die allerdings erneute Öffnungen nach sich zogen, da die AGF selbst darum bemüht war, als gleichberechtigter Partner in der Allianz agieren zu können. Dieses komplexe Zusammenspiel von kooperativen und kompetitiven Handlungsmodi resultierte schlussendlich in einer Institutionalisierung und Festigung der Allianz.

## Introduction: Cooperation, Competition and Their Effects on Processes of Institutionalization

The political thinking of the Federal Republic of Germany in the 1960s and early 1970s was characterized by a phase of planning euphoria and the desire for closer links between science and politics (Trischler 1990: 117–119; Orth 2011: 155–165). This in turn was shaped both by the perception of living in an age dominated by science and technology and by the hope of being able to solve general social problems with the help of scientific policy advice (Rudloff 2004: 13–17; Schimank 2009: 232–234; Seefried 2010: 111–116). Various ministries, as well as the parliament,^1^ increasingly consulted experts and scientific findings were incorporated into the development of political programs.^2^ Participation in political decision-making processes enabled scientific stakeholders to actively shape politics. Consequently, although the spheres of science and politics follow their own internal logics, the two are closely interrelated and serve as resources for each other (Ash 2002, 2010: 11–18, 2016; Bourdieu 1992: 49–81). Since the 1960s, the Federal Republic of Germany has increasingly seen both a scientification of politics and at the same time a politicization of science—and thus an even closer intertwining of those areas. These two processes are by no means mutually exclusive but can be regarded as complementary, sometimes even mutually dependent (Rudloff 2004: 16–17; Weingart 1983, 2005: 11–35). Consequently, a quantitative increase in scientific policy advice and an increasing institutionalization of different advisory formats can be observed during this time (Rudloff 2005: 1–19).

It is not a coincidence that this period also witnessed the informal establishment and gradual institutionalization of a committee^3^ that remains influential in German science policy up until today: the Alliance of Science Organisations.^4^ According to its own statements, the Alliance “brings together the top research organisations in Germany” (Hochschulrektorenkonferenz 2020). Its members regularly meet to discuss key issues of science policy or research funding. Furthermore they coordinate their financial interests, as all of them are funded by the public sector.^5^ However, these institutions are extremely heterogeneous with regard to their basic legal status, their internal organization and their function within the German science system: Representatives of (non-)university research, research funding agencies and, finally, scientific advisory bodies come together in the Alliance and raise different concerns in the joint meetings. Hence, the question arises as to how access to and cooperation within the Alliance was (and is) regulated. In order to examine this question, this study focuses on the 1970s and 1980s, which represented a formative period for the Alliance. During these years, the Alliance underwent an initial phase of formalization of its cooperation. Simultaneously it had to deal for the first time with serious destabilization in its internal organization caused by the admission of a new member.

One might assume on the one hand that the members of the Alliance are in direct competition with each other for the limited financial resources of the federal and state governments. In addition, there are other areas of competition, such as for the best scientists or for top positions in rankings (Nickelsen 2014; Nickelsen & Krämer 2016; Brankovic et al. 2018). In this respect, the decision by the science organizations to act cooperatively appears all the more surprising, especially since their goals may overlap in many areas, which may further encourage competition and hinder cooperation (Simmel 1986; Vogt 2015: 193–195; Werron 2011; May 2018). On the other hand, the name “Alliance of Science Organisations” suggests that the committee’s main purpose is to foster close cooperation.

When defining cooperation and competition with a focus on the participants’ respective aims, the interconnection between these two modes of interaction becomes visible: In both cases, the players are striving for the same goal. While cooperation implies that the actors can only achieve their common goal when working together, competition implies a negative interdependency: Competitors can only achieve their aim if others fail to do so (Nickelsen 2014; Nickelsen & Krämer 2016; Soutschek & Nickelsen 2019; Simmel 1986). Yet in practice, we can observe the intertwining of these two modes of interaction within the Alliance of Science Organisations as it is neither a purely cooperative committee, nor do its members only compete with each other. Instead, the Alliance is a particularly good illustration of the instability of both modes of action as well as of the tense interconnection between cooperation and competition (Nickelsen & Krämer 2016). The internal cooperation between the members of the Alliance is subject to constant negotiation processes and is characterized by considerations as to whether a goal can be achieved better by acting cooperatively or individually. As a committee, the Alliance of Science Organisations, in turn, operates in other competitive constellations, in which, for example, access to resources of power and influence in setting the agenda for German science policy are of great importance. The joint appearance of the presidents and chairpersons of the most influential science organizations has always made their statements particularly effective, which is why the Federal Minister of Research invited the Alliance to meetings on a regular basis within the so-called Presidential Circle.^6^ The Alliance thus is not only an intermediary actor of self-governing research, but also a science policy advisory body, which primarily serves the preliminary and informal coordination of interests between the science organizations and the ministry regarding structural changes in German research policy. This privileged access to the relevant political stakeholders in the field of science and research policy in turn makes membership in the Alliance of Science Organisations highly attractive to those standing outside. Consequently, the Alliance operates in the field of tension between cooperation and competition. The committee regularly has to face the question of whether the involvement of other actors strengthens its position, or if the inclusion of further perspectives in the joint discussions weakens its voice. Hence, this article examines how decisions about potential inclusion or exclusion were made: who decided on membership in the Alliance of Science Organisations and who determined the opening or closing of the committee? Which actors were regarded as capable of satisfying the membership requirements and therefore were allowed to enter this elite circle? And how do these processes of exclusion and inclusion affect the inner workings of the Alliance? On a broader conceptual level, two important questions arise: In what way are cooperative and competitive practices interrelated in the Alliance, the central coordinating committee of the science organizations? And how did these social practices influence the German science system on an institutional level? By exploring these central topics, this article will contribute to discussions on the dynamics of cooperation and competition, focusing in particular on their social and institutional dimensions.

The first part of this essay aims to clarify the extent to which the Alliance of Science Organisations can be considered an institutionalized coordination and advisory body, despite the informality of its meetings. In a first step, the development and progressive formalization of the Alliance in the 1970s and 1980s will be examined, which has received little attention in historical and social-science research so far. In doing so, the institutionalization of the meetings of the so-called Presidential Circle will be discussed as a corporatist attempt to shape German science politics. The process of institutionalization formalizes and consolidates structures, and thus regulates inclusions and exclusions, also regarding participation in political advisory bodies (Wenninger et al. 2019: 38–40).

In the second part, a specific case study of the practices of inclusion and exclusion is analyzed. This section concerns the relations of the Association of the Major Research Centers (*Arbeitsgemeinschaft der Großforschungseinrichtungen*, AGF)^7^ to the established German science organizations. The inclusion of this association, which later developed into the Helmholtz Association, represented the first enlargement of the Alliance. For the analysis of the Alliance’s complex relation to the AGF, this article will draw on some historical and social-science studies on the development of the German research system in general as well as on studies on the history of the AGF in particular.^8^ However, the Alliance of Science Organisations has so far only appeared on the margins of studies dealing with individual science organizations that are members of the Alliance.^9^ For this reason, this paper is based primarily on previously unexamined archival collections on the Alliance and the Presidential Circle.^10^

The aim of this paper is to shed light on the social and institutional level of cooperation in the Alliance of Science Organisations and, in doing so, to examine the interactions of the science organizations within the committee, as well as in relation to the central actors of the Federal Government in the field of science policy. This article argues that the Alliance can be understood as a structurally rather conservative committee which initially often perceived changes as disrupting its established patterns of interaction. In order to restabilize the situation, exclusionary and competitive practices were used by the members of the Alliance (Stucke 2016; Hohn 2016). Thus, this article aims to analyze how practices of inclusion and exclusion have been deployed by the stakeholders of the Alliance to meet different expectations and to balance a variety of interests at the same time. Only this complex interplay of cooperative and competitive practices—so the hypothesis—finally resulted in the institutionalization of the Alliance.

## Institutionalization of the Alliance of Science Organisations

For a long time, the Alliance has been perceived as a highly informal organization. The committee has been described by external observers as some sort of “secret gatherings of the Knights of the Roundtable” or as a club “that attempts to pull strings behind the scenes” (van Bebber 2011: 35). Although the Alliance has remained a rather informal committee up to this day with no office of its own, no permanent staff or press department and an external appearance that is relatively restrained, there have been signs of a gradual institutionalization of this cooperative committee since the 1970s.

### Formalization of Cooperation

The beginnings of the Alliance of Science Organisations date back to the late 1950s, when the West German Rectors’ Conference (WRK)^11^ and the German Research Foundation (*Deutsche Forschungsgemeinschaft*, or DFG) had an intensive exchange on the plans of the federal and state governments to create the German Science and Humanities Council (*Wissenschaftsrat*, or WR) and tried to influence its design (Bartz 2007: 23–49; Orth 2011: 100–106; Stamm 1981: 202–219; Stucke 2006: 248–252).^12^ At the same time the Federal Government had become active in the funding of research, as indicated by the creation of the Federal Ministry for Nuclear Affairs (*Bundesministerium für Atomfragen*, BMAt) in 1955 and the transformation of the latter into the Federal Ministry for scientific Research (*Bundesministerium für wissenschaftliche Forschung*, BMwF) in 1962. The creation of these bodies changed the dynamics in the German science system and should finally lead to the establishment of the Alliance of Science Organisations: In response to efforts of Atomic Minister Siegfried Balke to convince the presidents of the Max Planck Society (MPG), DFG and WRK to support his plan to create a Federal Research Department, these three science organizations moved closer together in order to protect themselves from any influence by the ministry in terms of regulating or controlling the content of research (Orth 2011: 100–116; Stucke 1993: 54–67; Stamm 1981: 225–252).

At a meeting of the Senate, Adolf Butenandt, president of the MPG, explained that one should “not take a position on the question of whether Germany required a special ministry for research individually” but rather seek out cooperation with the DFG and the WRK.^13^ Subsequently, the three science organizations, the MPG, DFG and WRK, continued their meetings from 1962 onwards with the involvement of the WR to discuss “foundational questions about science policy on a regular basis.”^14^ The joint decision to formalize their hitherto irregular meetings marks the birth of the Alliance of Science Organisations and can be understood as a reaction to the expansion of the federal government in the field of science policy (Schimank 1995: 106–111).

**Fig. 1:**
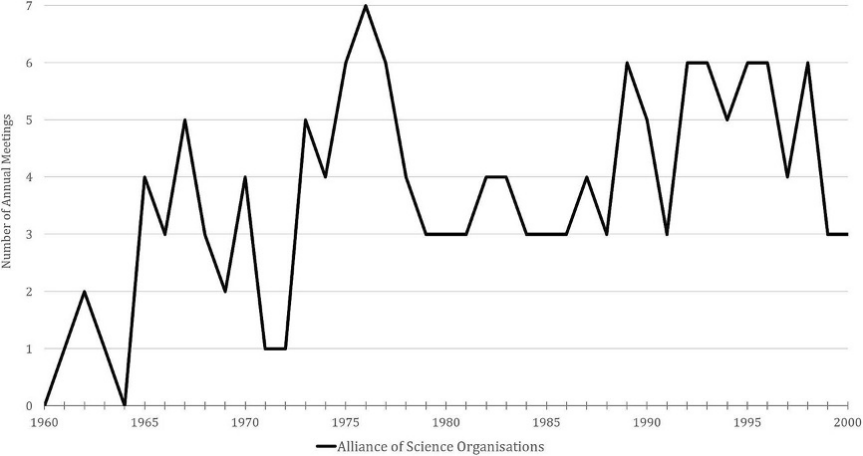
Number of annual meetings of the Alliance, 1960–2000. The compilation of the meeting dates is based on the information on the individual meetings in AMPG, II. Abt., Rep. 57 and DFGA, AZ 02219-04

The emergence of a new external negotiating partner in the federal government resulted in the first consolidation of this informal group, which had previously met mainly on the occasion of other meetings or at a joint dinner. A look at the frequency of the Alliance’s meetings over a longer period of time (Fig. 1) confirms the impression of a slow institutionalization process beginning in the 1970s, as their meetings became increasingly regular during these years. Officially, these meetings were referred to as “Meetings of the Presidents, Chairpersons and General Secretaries of the Science Organizations,”^15^ but the shorter name “Holy Alliance” or “Alliance” quickly became common among its members (Klofat 1991). The reference to the Holy Alliance founded in 1815 by Russia, Prussia and Austria, which had committed itself to mutual loyalty and cooperation despite all differences in political and confessional orientation was surely not accidental (Fehrenbach 2008: 126–135; Pyta 1996).

### Institutionalization of the Presidential Circle as an Advisory Body on Science Policy

For its part, federal politics reacted to the formation and consolidation of the Alliance and sought to exchange views with the presidents of the science organizations. After taking office, the first Federal Minister for Scientific Research (BMwF), Hans Lenz, contacted the presidents of the science organizations and met with them occasionally.^16^ However, it was his successor, Gerhard Stoltenberg, who intensified the relations between his department and the self-governing organizations with the aim of “regularly discussing the problems of science.”^17^

The establishment of regular consultations between the ministry and the presidents of the Alliance can also be understood as the formalization of their consultative role in processes of political decision-making.^18^ The Alliance benefited from the fact that they had already exchanged views on research policy issues with other ministries, including the Atomic Ministry, even before the BMwF was founded and had thus established the corporatist approach of mediating interests.^19^ To a certain extent, the self-governing organizations also benefited from the absence of a pre-defined political program or instruments in matters of research funding, which further encouraged the close coordination between politics and science. Through the joint efforts of the presidents of the self-governing organizations, the Alliance was able to intervene in its political environment and influence the shaping of German research policy (Braun 1997: 222–234; Stucke 1993: 54–67).

Consequently, Stoltenberg strengthened the already existing corporatist structures of German science policy by introducing regular consultations. At the same time, he created a framework for this informal exchange for the first time with the so-called “fireplace rounds” (*Kaminrunden*), as the meetings of the Presidential Circle were referred to in the 1960s and 1970s (Trischler & vom Bruch 1999: 87–92; Weßels 1999: 87–96). In 1966 Stoltenberg described the meetings with the presidents of the Alliance as an “extensive exchange of opinions,” which he hoped would continue “in casual succession.”^20^

The informal nature of the talks was to remain the defining characteristic of the Presidential Circle for a long time to come (Patzwaldt & Buchholz 2006: 463; Schimank 1995: 130–132). In 1974, for example, the staff of the Federal Ministry of Research and Technology (*Bundesministerium für Forschung und Technologie*, BMFT), which had been created in 1972, noted on behalf of minister Horst Ehmke, who wanted to renew the meetings with the presidents, that the Presidential Circle was an “informal discussion group.”^21^ Ehmke himself emphasized in the invitation to the presidents of the Alliance that he saw the joint talks as an opportunity “to discuss questions of research and technology policy in an intimate setting.”^22^

In 1978, Volker Hauff, then Research Minister, praised the presidents’ “great pool of experience,” which would find its way into the “elaboration of research policy.”^23^ Hauff’s statement reveals for the first time a certain commitment—albeit a vaguely formulated one—to the issues the group discussed together. The 1970s were characterized by frequent changes at the top of the BMFT. Despite the fact that most ministers remained in office for no more than two years,^24^ a trusting relationship began to develop between the ministry and the Alliance during the phase of the Social-Liberal Coalition (1969–1982). The high level of personnel continuity among the ministry officials who valued the presidents of the science organizations as reliable partners in questions of research policy proved beneficial. A briefing of the new minister, Andreas Bülow, in 1980 illustrates the extent to which ministry officials valued the Alliance. Bülow was not very familiar with the field of research policy and the staff tried with the utmost effort to convince him of the high importance of the Alliance as an “informal advisory body” and to prepare him for the meeting with regard to its contents.^25^

Two years later, on the occasion of another change at the top of the Research Ministry, the importance of the meetings with the presidents of the Alliance was outlined even more precisely: The Presidential Circle was now characterized as an “advisory committee of the BMFT that met roughly two times each year.”^26^ In addition, the incoming minister Heinz Riesenhuber assured the presidents of the Alliance during a joint meeting of their role as “top councilors,” through whom “the essential views of the science organizations would be realized in research policy.”^27^ Riesenhuber, a chemist and proven expert on research policy, would remain in office for more than ten years and had great interest in strengthening the ministry’s relationship to the presidents of the Alliance.

**Fig. 2:**
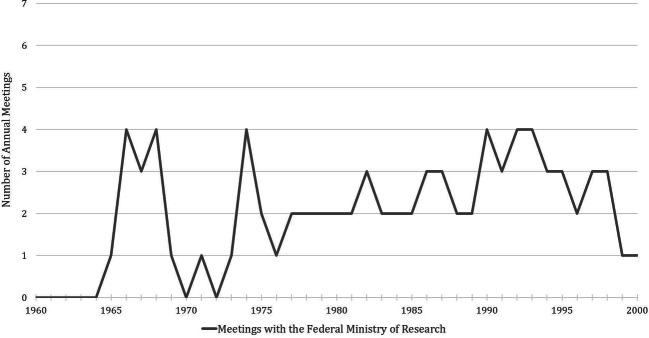
Number of annual meetings with the Federal Ministry of Research (Presidential Circle), 1960–2000. The compilation of the meeting dates is based on the information on the individual meetings in AMPG, II. Abt., Rep. 57, DFGA, AZ 02219-04 and BArch, B 196

The contacts between the ministry and the Alliance became closer in the second half of the 1970s and the early 1980s. This resulted in an increasing regularity of their meetings (Fig. 2), which accelerated the institutionalization of the Presidential Circle in the following years. In addition to the increasing regularity of the meetings, one can find further signs of an institutionalization beginning in the mid-1970s: For example, the BMFT started to send out official invitations with a preset agenda for each meeting. The participants also came carefully prepared for the individual items to be discussed in these meetings. In the ministry, the respective (sub-)heads of the departments prepared detailed consultation notes for the Federal Minister of Research on the planned topics in advance. In the Alliance, the preliminary discussion of the Presidential Circle and the coordination of a common line towards the ministry became a fixed part of the internal agenda.^28^

In comparison to the Alliance of Science Organisations, the meetings of the Presidential Circle were of a more official nature. In fact, the Presidential Circle can be considered a gathering of some of the most important stakeholders from the two areas of science and politics. Therefore, the formalization of cooperation and internal workflows on both sides began earlier while also proceeding more quickly. By the early 1980s, the perception of the Presidential Circle—and thus also of the Alliance—had changed massively. Originally conceived as a casual discussion forum, it developed into an institutionalized, in-house advisory body of the BMFT, whose advice found its way into the ministry’s research policy programs as a matter of course.

However, it is difficult to measure the influence of the Alliance in terms of specific successes or cases in which the presidents put their mark on decisions regarding science policy. Instead, the joint discussions were characterized by an open and trusting exchange of ideas: The minister usually gave a short overview of his plans and sought the science organizations’ advice. In this way, the Alliance was able to contribute its ideas and priorities before specific political programs were elaborated.^29^ The BMFT trusted the presidents of the science organizations in matters of science policy and regularly consulted them, for example, when developing general funding priorities for the next few years.^30^ Therefore, trust is a key term to describe the relationship between science and politics as represented in the Presidential Circle. The minister trusted the presidents of the Alliance to provide him with the necessary (and valid) information for his subsequent political actions, while the science organizations for their part trusted that the minister would act in their interest (Luhmann 2014; Frevert 2013). Furthermore, the ministry did not hesitate to ask the presidents to get in touch with other politicians or the public in order to support their common goals. Thus, one can conclude that the relationship between the ministry and the science organizations can be characterized as having been highly cooperative, although the Alliance was also dependent on the ministry for its funding.

Through their regular consultations in the Presidential Circle the Alliance had privileged access to the ministry. Moreover, the BMFT incorporated the Alliance’s proposals into the design of its research policy. This inclusion of the expertise of the Alliance’s members in political decision-making processes further enhanced the reputation of the science organizations that comprised the Alliance and thus contributed to consolidating their position at the top of the German science system.

## The Composition of the Alliance: Inclusion and Exclusion Using the Example of the Association of the Major Research Centers (AGF)

With the formalization of cooperation within the Alliance, its gradual institutionalization and recognition as a top advisory body by the Federal Ministry of Research, the committee was also given a higher profile in the political and scientific communities (Hohn & Schimank 1990: 401–413). This not only increased interest in its work, but also brought its composition into the focus of various actors. Questions of membership became a central issue of scientific prestige and power in research politics.

This resulted—sometimes through indirect pressure from outside, as will be shown below—in the inclusion of previously excluded science organizations. At the same time, however, the Alliance tried to keep the circle of its members as small as possible in order to preserve its own ability to act. This necessitates a tension-filled simultaneity of opening and closing tendencies, which will now be examined in more detail using the example of the relationship between the AGF and the Alliance of Science Organisations.

### Context: The Association of the Major Research Centers (AGF)

In 1970, the ten major research centers existing in the Federal Republic of Germany founded the Association of the Major Research Centers* (Arbeitsgemeinschaft der Großforschungseinrichtungen*, AGF) (Hoffmann & Trischler 2015: 13–21; Szöllösi-Janze 1990b: 149–154).^31^ The foundation of the AGF thus coincided with a phase of increasing resource scarcity resulting from the economic crisis which began in the 1970s. Although the concrete effects may have varied from case to case, financial growth slowed down noticeably and, in some cases, even stagnated completely (Hohn & Schimank 1990: 406–413). The experience of the economic crisis and budget tightening fostered more competitive behavior among the science organizations, especially when dealing with a newly formed stakeholder, whose budget from federal funds was many times higher than that of the other Alliance members. In these tense times, the major research centers began to consolidate their identity as a separate type of non-university research, which the other science organizations observed with a critical eye. However, over the subsequent years the AGF was not able to fully realize its political ambitions: Until the 1980s, the chairman only had an office with minimal staff which he administered on a part-time basis; the individual research centers continued to remain legally independent. Thus, the powers of the chairman of the AGF in internal matters were initially not particularly far-reaching (Hoffmann & Trischler 2015: 13–24). This limited capacity for action was of course apparent to his colleagues in the Alliance and so they mockingly asked whether the AGF’s chairman must first seek permission from his association before joining the Alliance.^32^

### Inclusion Through Politics: the Admission of the AGF into the Presidential Circle

In 1974, two years after the division of the department for education and science (BMBW) from the one for research and technology (BMFT), the Minister of Research Horst Ehmke decided to intensify the relations between the science organizations and his department. Furthermore, he also enlarged the group of participants and invited the chairman of the AGF to join (Ritter et al. 1999; Szöllösi-Janze 1990a; Szöllösi-Janze & Trischler 1990; Hoffmann & Trischler 2015).^33^ The members of the Alliance initially showed reservations towards the newest negotiating partner within the Presidential Circle. Nevertheless, the ministry had no direct influence on the composition of the Alliance and on whom the science organizations invited to their internal meetings, even though the integration of the AGF into the Alliance was definitely in the ministry’s interest (Szöllösi-Janze 1990b: 155–156, 302–310). One year later, federal politics confirmed its position towards the AGF and thus strengthened its position within the German science system: Since the establishment of the WR in 1957, only the WRK, the DFG and the MPG had been entitled to send the Federal President a list of suggestions for appointments to this council. In 1975, following the amendment of the administrative agreement, the AGF was also granted the right to submit suggestions for appointments to the WR (Bartz 2007: 123–127; Röhl 1994: 11; Rusinek 1996: 752).

### The AGF and the Alliance: Reluctant Inclusion

This amendment now required a vote between the Alliance and the AGF regarding the nominations for the WR. The organizations entitled to suggest appointments needed to draw up a joint list of people suited to fill the vacant positions to be submitted to the Federal President (Röhl 1994: 11–13).

At first, the members of the Alliance and the AGF discussed the appointment lists prepared in the respective subcommittees separately. Within the Alliance, the presidents of the WRK, DFG and MPG finally agreed on a common list of names, while the DFG took over the task of coordinating this list with the AGF.^34^ This procedure proved to be impractical and was therefore discussed at a meeting of the Alliance in 1976: MPG president Reimar Lüst remarked with a certain displeasure that the Alliance would have to consult with Karl Heinz Beckurts, chairman of the AGF, on the nominations for the Scientific Commission of the WR.^35^ His phrasing indicated that the coordination of the list of proposals with the AGF was initially met with disapproval because this process prolonged the procedure of appointing new members to the WR.

Consequently, DFG president Heinz Maier-Leibnitz suggested inviting Beckurts as a guest to their joint meetings.^36^ However, the invitation of the AGF’s chairman to the Alliance’s meetings did not come about as a result of internal conviction or even recognition of the growing importance of the AGF. Rather, this decision was shaped by a certain necessity and pragmatism that aimed to avoid unnecessary and additional work. It is also remarkable that Beckurts was initially not intended to become a member of the Alliance. While the other participants were addressed as representatives of their respective organizations, the members of the Alliance insisted on inviting Karl Heinz Beckurts explicitly as a guest and not as an official representative of the AGF. The fact that the Alliance nevertheless decided to invite Beckurts to the joint meetings was probably helped along by the high scientific reputation he enjoyed as a very successful nuclear physicist. He was furthermore the chairman of the KfA Jülich and was thus certainly one of the most influential science managers within the AGF (Rusinek 1996: 739–740, 752–754). Consequently, Beckurts benefited from his personal and professional authority, since the guest status that the Alliance granted him was directly linked to his person (Bourdieu 1992: 49–81). However, this did not change the Alliance’s concerns about including the AGF as an organization. Their reservations were fueled by different factors: The members of the Alliance considered the work carried out in the major research centers to be to a certain extent research commissioned by the state. Hence, they feared that the inclusion of the AGF would undermine their claim of autonomy. Furthermore, the Alliance was afraid of a growing competition in matters of association policy due to the fact that the major research centers had founded the AGF and thus started acting cooperatively. This in turn, put the carefully balanced distribution of power between the science organizations and the division of their fields of activity to the test. At the same time, the major research centers remained legally independent. That is why the AGF was regarded as an association with very little authority. Consequently, the question was raised as to whether the AGF would be able to act as a negotiating partner of equal status (Szöllösi-Janze 1990b: 154–156, 302–303). The AGF nonetheless interpreted the invitation as a first step towards its official recognition by and inclusion in the Alliance. Membership in the Alliance gave the AGF the opportunity to participate in the processes of consensually influencing political decisions. The committee was perceived as a forum that allowed the science organizations to lend their interpretations a sense of validity which went beyond the sensitivities of a single institution and therefore was accepted by the political authorities and taken into considerations. Herwig Schopper, chairman of the AGF beginning in 1977, announced at a general meeting that “the conversations in the so-called “Alliance” have flourished” (Szöllösi-Janze 1990b: 308).^37^ Indeed, during Schopper’s term of office, not only the “coordination with other science organizations, such as the Alliance” was intensified, but also “bilateral actions” were started, for example in the form of a joint recommendation with the WRK.^38^ Internally, the AGF had the feeling that they had succeeded in further improving their association.^39^

### Exclusion Despite Opening

Although the Alliance had slowly opened up to the AGF since 1976 due to indirect external pressure from the Federal Ministry of Research, this transformation took place neither in a tension-free environment nor in a linear manner.

Even in the 1980s, more than four years after the first invitation of its chairman to the exclusive meetings of the Alliance, the AGF continued to see itself being pushed into an outsider position. Thus, Gisbert zu Putlitz, the new chairman of the AGF, noted in an internal memo regarding the nomination of new members for the WR in 1981:

The Federal President receives information about suggestions for the coming appointments to the German Council of Science and Humanities. The documents used by Mr. Lüst, Mr. Turner and Mr. Seibold are not in my possession.^40^

Zu Putlitz therefore experienced a similar dynamic to the one Herwig Schopper had faced a few years earlier: While the relationship between the AGF and the Alliance seemed to have stabilized under his predecessor—especially towards the end of his term of office—the situation suddenly changed again when the office was handed over. It becomes clear that the reputation of the office-holder among the established science organizations was directly linked to the respective person and was not automatically passed on to his successor (Szöllösi-Janze 1990b: 306–310). Instead, the successor remained highly dependent on his own personal and professional prestige being recognized by the Alliance. Especially at the beginning of his term of office, he was initially confronted with their reservations about the organization he was chairing. In zu Putlitz’s case, for example, this was expressed by the fact that he did not receive circulating documents in the run-up to joint meetings. Moreover, the founding members of the Alliance did not hesitate to express their considerable reservations in the joint meetings.^41^

However, this rejection was not only expressed in a subtle way in the internal circle. On the contrary, his exclusion was also openly displayed. For example, zu Putlitz reported the following from a meeting of the Presidential Circle when the budget situation of the Federal Ministry of Research and funding cuts were discussed:

The BMFT reports that the budget for personnel and expenses at the major research centers will be reduced […] Lüst und Seibold used this opportunity to unleash complaints about the quality of the major research centers.^42^

The situation described is remarkable in that the presidents of the MPG and DFG did not hold back their criticism of the AGF and its work in the presence of the Federal Minister of Research and his state secretaries, as well as in the presence of the president of the AGF. The question of the distribution of financial resources was still incendiary in the early 1980s and had a destabilizing effect on the fragile cooperation with the AGF. In times of general funding shortages, the MPG and DFG perceived the major research centers as competitors for state resources and feared losses for their own organizations. Although the AGF had already been officially accepted into the circle of the so-called Holy Alliance at this point in time, its founding members made the newcomer aware of the limits of acceptance and its position as an outsider. Completely excluding the AGF from the joint negotiations was, however, not a viable alternative since the Alliance did not want to offend the Ministry of Research.

### Gradual Acceptance

The other science organizations perceived the rise of the AGF and its establishment as a new pillar in the German research system as a threat to their own position. Another partner that brought its own wishes and ideas into the negotiations threatened to reduce the influence of the long-standing members of the Alliance. These dynamics had also become apparent to the members in the Alliance—with the MPG taking the lead in this respect—and to the AGF. In order to defuse the situation, Reimar Lüst, president of the MPG, finally invited his colleague zu Putlitz, chairman of the AGF, to a discussion in January 1982:

At the outset of this conversation, Mr. Lüst expressed his concerns and fears that a sort of competition might arise between the major research centers and the Max Planck Society that would be mutually unbeneficial. The special status of the Max Planck Society must be recognized.^43^

Fears that the MPG’s own position—whether in financial or scientific terms or in matters of science politics—could be threatened by the AGF seemed to influence the MPG’s behavior. However, the AGF could not be completely ignored, especially given the interest of the Federal Ministry of Research in their involvement, which is why the MPG finally opted for a joint consultation. Direct competition between the MPG and the AGF was now apparently regarded as a problem. The MPG perhaps hoped it would be easier to reach its goal in close cooperation with the AGF, especially when dealing with the ministry.

Zu Putlitz for his part was well aware of the importance of a cooperative relationship to the MPG as one of the leading institutions within the Alliance and tried to dispel the concerns immediately by emphasizing that he saw “no conflict at all” concerning “the special status and special rights of the Max Planck Society.”^44^ Instead, he emphasized that they shared a common goal, which could only be achieved together.^45^

Whether this conversation was the trigger for the stronger integration of the AGF into the Alliance cannot be said with certainty. However, from about 1982 onwards, various episodes of inclusion can be observed with regard to the AGF.

**Fig. 3:**
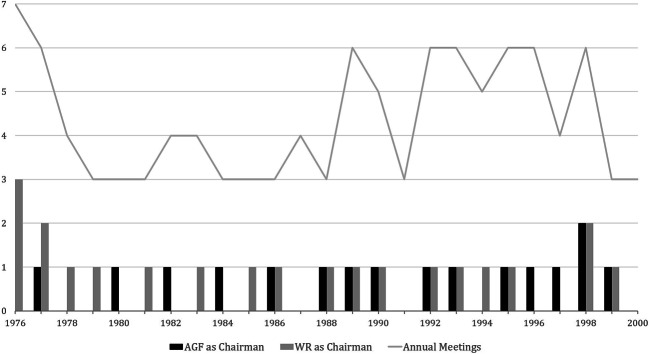
Comparison of the Association of the Major Research Centers (AGF) and the German Council of Science and Humanities (WR) regarding the chairmanship of meetings of the Alliance of Science Organisations, 1976–2000. The comparison of chairmanship of meetings is based on the information on the individual meetings in AMPG, II. Abt., Rep. 57, DFGA, AZ 02219-04, DFGA, AZ 0224 and AdHRK “Allianz und Präsidentenkreis”

A look at the chairs at the meetings of the Alliance of Science Organisations (Fig. 3) shows that in the 1970s the AGF only chaired one single meeting, whereas in the 1980s it chaired one meeting roughly every two years. Compared to the total number of annual meetings, this at first glance may not seem like convincing evidence for inclusion.

However, if we compare the data with the chairs of the WR, one of the founding members of the Alliance, a different picture emerges. The first feature that stands out is the discrepancy in the 1970s. During this period, the WR organized at least one, sometimes even two or three meetings a year. In the 1980s, the bars gradually even out.

**Fig. 4:**
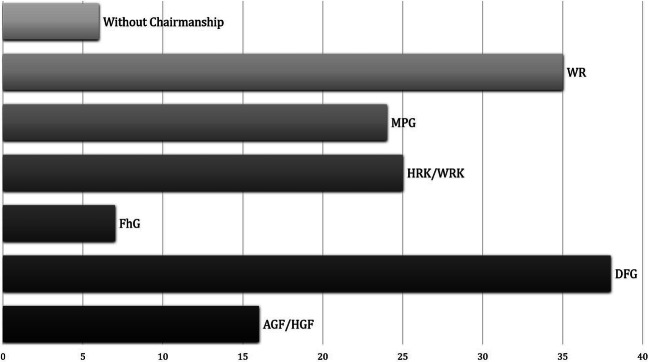
Total number of chairs of the meetings of the Alliance of Science Organisations, 1961–2000. The comparison of chairmanship of meetings is based on the information on the individual meetings in AMPG, II. Abt., Rep. 57, DFGA, AZ 02219-04, DFGA, AZ 0224 and AdHRK “Allianz und Präsidentenkreis”

Since the WR chaired 35 meetings of the Alliance between 1961 and 2000,^46^ this development shows that the AGF was able to establish itself within the Alliance—at least in regard to the chairmanship (Fig. 4). By taking over the chairmanship, the AGF was therefore in a position to set the main focus of the meeting and to significantly shape the agenda in a way that went beyond mere participation.

In the following years, the AGF was also increasingly involved in the elaboration of joint statements, for example in a 1986 statement concerning deregulation in non-university research.^47^ Even the MPG, which did not hold back its criticism of the AGF at the beginning of the 1980s, showed itself to be increasingly cooperative: When the members of the Alliance discussed a change in tax law that AGF and FhG wanted to initiate together, the MPG announced that it would refrain from objecting to the plans even though it did not correspond with its interests. In return for this favor, the MPG demanded that the other members of the Alliance sign the MPG’s declaration on collaborative research.^48^

The atmosphere in the Alliance had obviously changed: Now the founding members had granted the AGF the right to act as an equal partner. A further indication of the AGF’s inclusion into the Alliance during this period is its leadership in the preparation of joint statements. To cite just one prominent example, the AGF coordinated joint actions and public statements of the Alliance regarding animal protection in the early 1990s. 49While each of the science organizations had their own priorities, the founding members entrusted the AGF with managing the statement. This close involvement in the workings of the Alliance shows that the AGF was finally perceived as an equal-ranking member of the Alliance. After all, the statements published by the Alliance were public actions, and thus reached a select audience in science and politics.

## Conclusion

Examining the practices of inclusion and exclusion allows us to better understand the internal dynamics of the Alliance and trace the constant balancing act between cooperation and competition. This also enables us to draw conclusions regarding the social dimension of cooperation between the science organizations in the Alliance. In this view, we can observe the simultaneity of modes of action that may appear contradictory at first glance: practices of inclusion and exclusion coexist, as do modes of cooperation and competition. Since the Alliance is an informal committee that brings together the most important science and research organizations in Germany, it may initially appear as being solely designed for cooperation and thus for including various players. This cooperation, however, also requires an exclusionary element with respect to other institutions that are not represented in the Alliance and, consequently, with respect to their interpretations and perspectives. Investigating the history of the Alliance in the 1970s and 1980s also showed that the internal relationships between the members were by no means exclusively cooperative; questions of hierarchy and competition for resources shaped their collaboration in many ways.

The formalization of cooperation between the science organizations in the Alliance by fixing a meeting schedule and carefully preparing joint meetings came as a reaction to the growing attempts of the federal government to shape science policy since the 1960s. Since its foundation, the Federal Ministry of Research has tried to maintain a close exchange with the presidents of the science organizations assembled in the Alliance. The ministry’s aim was to consult with the science organizations on important issues in order to ensure the legitimacy of its decisions regarding research policy in the eyes of a wider public. This also meant that the Alliance was officially accepted by the federal government as being a reliable committee of experts, which the minister and his officials trusted in questions of research policy. Moreover, the politicians also hoped that the decisions achieved in agreement with the Alliance would be implemented internally by the science organizations. The relationship between the participants in the Presidential Circle can be described as an exchange of ideas in an atmosphere of great trust. These meetings offered the Alliance the opportunity to actively shape German science policy together with the ministry. However, the nature of these discussions changed in the course of the 1970s, when the Alliance became explicitly recognized as the BMFT’s advisory body. Consequently, the institutionalization of the cooperative consultations resulted in the active inclusion of scientific expertise from the political side.

At the same time, changing constellations in the German science system made it necessary to admit new players to the Alliance and thus resulted in the opening of this committee. Probably the most tension-filled inclusion of a science organization in the Alliance was its first expansion: The admission of the AGF in 1976. As an independent player in science policy, the AGF was a comparative newcomer who was invited to the Presidential Circle by the Federal Ministry of Research. The inclusion of the AGF into the joint negotiations took place in a time of scarce resources. The uncertainty about the allocation of financial resources and about the role of the AGF in German science politics resulted in different elements of exclusion.

The initiative of federal politics to grant the AGF the right of nomination for members of the WR and its admission to the Presidential Circle forced the Alliance to pursue closer coordination with the AGF. This finally led to the invitation of the AGF’s chairman to the meetings of the Alliance. In theory, the members of the Alliance themselves decided on the composition of their committee. However, the Alliance could not completely ignore the demands of external parties. In the following decades, further steps towards the inclusion of the AGF took place. Among other changes, the AGF began to assume leadership of meetings and coordinate cooperative actions with other members.

Nonetheless, this process of inclusion was by no means a linear development. This is hardly surprising, since the inclusion of the AGF disrupted the well-established cooperation of the hitherto unchallenged four large science organizations. While mostly external actors—such as the Federal Ministry—suggested including certain organizations in the Alliance, its members at times acted to exclude newcomers and limit their influence. After all, accepting a new partner in the Alliance implied an additional perspective that had to be included in the negotiation process. In turn, new members threatened to weaken the positions of the four founding members. This initially had a destabilizing effect on the carefully balanced relationship of power in the Alliance (Bourdieu 1985: 72–81). The more or less subtle attempts of a partial exclusion of the AGF on different levels can therefore be understood as an attempt to stabilize the prevailing distribution of power in the German science system. The Alliance’s reaction to the change in its internal dynamics spurred by the inclusion of the AGF was more than a mere demonstration of power for its own sake: Membership in the Alliance also implied the possibility of shaping German science policy in a corporatist way. As a result, the status of new members remained dependent on the established science organizations of the Alliance; their assessments of whom they considered capable of participating in this circle and, consequently, of supporting their common interests in a cooperative manner proved decisive (Goffman 1969: 95–97).

The moments of exclusion in certain areas of cooperation which the AGF had to face should be interpreted with regard to their symbolic dimension. By including and excluding other science organizations, the Alliance began to increasingly define itself, its mission and its elite membership; these developments finally led to the institutionalization of the committee. The analysis of the internal relationships within the Alliance has shown that inclusion at one level could trigger exclusion at another level, sometimes involving the same players. Cooperation can therefore be understood as an unstable mode of interaction from which actors may withdraw at any time (Nickelsen 2014: 355–357).

## Acknowledgements

I would like to thank the anonymous reviewers and the editorial staff of NTM for their helpful suggestions on the first version of this essay. I am especially grateful to Helmuth Trischler for his help and constructive suggestions during the revision of the manuscript. Furthermore, I also thank the members of the DFG Research Group “Cooperation and Competition in the Sciences” (FOR 2553), who discussed an early draft of the text with me, and the members of the Research Program “History of the Max Planck Society” (Max Planck Institute for the History of Science, Berlin). Finally, I wish to thank Anna Lehner, Ramona Pohlmann and Fabienne Will for their valuable comments and suggestions and Moritz Schlenker for his support during the research process.

## Footnotes

1 For example, in the form of commissions of enquiry on issues such as future nuclear energy policy (1979–1983) or on the opportunities and risks of genetic engineering (1984–1987).

2 For example, the results of the working group “In Vitro Fertilisation, Genome Analysis and Gene Therapy” (1984–1985) convened by the Federal Minister of Justice and the Federal Minister of Research and Technology were incorporated into the drafting of the Embryo Protection Law (Bogner 2006: 486).

3 Due to its informal qualities, it is difficult to define the Alliance of Science Organisations in Germany and its work conceptually. When speaking of the Alliance’s internal organization and of the way in which the members cooperate with each other the term “committee” is sometimes used. The term “advisory body” is also used to describe its relationship to politics. Both terms were coined by the actors themselves during the time span on which this paper focuses and therefore seem promising for characterising the Alliance in more detail. For the use of the German term *Gremium*, which can be translated with the English term “committee” see e.g. BArch, B 388/1. Interner Vermerk der AGF zur Sitzung der Allianz am 10.06.1982; Archiv der HRK (AdHRK), Allianz und Präsidentenkreis, Bd. 4. Interner Vermerk der HRK über die Sitzung der Allianz am 28.08.1989; AMPG, II. Abt. Rep. 57, Nr. 613. Interner Vermerk der MPG über die Sitzung der Allianz am 03.03.1993.

4 Today, members of the Alliance include the Alexander von Humboldt Foundation (AvH), the German Research Foundation (*Deutsche Forschungsgemeinschaft*, DFG), the *Fraunhofer-Gesellschaft* (FhG), the German Academic Exchange Service (DAAD), the German Council of Science and Humanities (*Wissenschaftsrat*, WR,), the German National Academy of Sciences Leopoldina, the German Rectors’ Conference (HRK), the Helmholtz Association of German Research Centres (HGF), the Leibniz Association (WGL), and the Max Planck Society (MPG).

5 DFGA, AZ 02219-04, Bd. 11: Schreiben von D. Simon an G. Schettler vom 01.09.1989.

6 The German term is *Präsidentenkreis*. This term was introduced by the Federal Ministry of Research in the 1970s and was subsequently adopted by the Alliance. See BArch, B 196/16341. Interner Vermerk zur Einladung für die Hl. Allianz vom 01.02.1974.

7 The AGF was the forerunner of the Helmholtz Association of German Research Centres (HGF).

8 See for example Braun (1997), Fisch & Rudloff (2004), Hoffmann & Trischler (2015), Hohn & Schimank (1990), Mayntz & Scharpf (1995), Ritter et al. (1999), Stamm (1981), Stucke (1993), Szöllösi-Janze (1990a, b), Szöllösi-Janze & Trischler (1990).

9 A good example are the studies on the history of the *Fraunhofer-Gesellschaft* (Trischler & vom Bruch 1999), of the German Council of Science and Humanities (Bartz 2007), of the Leibniz Association (Brill 2017) and of the German Research Foundation (Orth 2011; Walker et al. 2013; Zierold 1968). Historical studies which focus on research organizations that only recently joined the Alliance, like the German Academic Exchange Service (Alter 2000) and the Leopoldina (Gerstengarbe et al. 2016) hardly ever discuss their cooperation within the Alliance.

10 This article is based on archival collections from the Archive of the MPG (AMPG) in Berlin and the Archive of the DFG (DFGA) as well as the Archive of the HRK (AdHRK), both located in Bonn. Additionally, archival collections from the Ministry of Research and Technology (BMFT), the German Chancellery (*Bundeskanzleramt*, BK) and the AGF, which can be find in the Federal Archive (BArch) in Koblenz make up a central part of the corpus of sources for this study. The different archival collections of the MPG, DFG, the ministry and the German Chancellery cover the time from the 1960s onwards, but none of these record groups is without gaps. The collection of the AGF starts in the 1970s, while the one of the HRK begins in 1980. These different archival collections include for example documents from the meetings of the Alliance and Presidential Circle, including internal minutes, extensive correspondence between members of the Alliance and internal reports on the cooperation within the Alliance. As all of the above-mentioned archival material is in German the following English quotations have been translated.

11 The WRK was the forerunner of the German Rectors’ Conference (HRK).

12 References to a coordination between the science organizations can be found in DFGA, AZ 6, Bd. 1. Entwurf über das Verwaltungsabkommen zum Wissenschaftsrat vom 15.06.1957; Ibid., Stichwortzettel der DFG zur Besprechung über den Wissenschaftsrat im Bundeskanzleramt vom 17.08.1957; Ibid. Schreiben von G. Hess an H. Coing vom 21.06.1957; AMPG, II. Abt., Rep. 60, Nr. 37.SP. Niederschrift der Sitzung des Senats der MPG am 11.11.1960.

13 AMPG, II. Abt., Rep. 60, Nr. 40.SP. Niederschrift der Sitzung des Senats der MPG am 06.12.1961.

14 AMPG, II. Abt., Rep. 60, Nr. 41.SP. Niederschrift der Sitzung des Senats der MPG am 09.03.1962.

15 See the headings of the respective minutes during the late 1960s and early 1970s, e.g. in AMPG, II. Abt., Rep. 57, Nr. 602, Bd. 1 und Bd. 2; AMPG, II. Abt., Rep. 57, Nr. 603; DFGA, AZ 02219-04, Bd. 1a.

16 See for example AMPG, II. Abt., Rep. 60, Nr. 44.SP. Niederschrift der Sitzung des Senats der MPG am 13.03.1963; AMPG, II. Abt., Rep. 69, Nr. 334. Auszug aus der Niederschrift der Sitzung des Wissenschaftlichen Rates der MPG am 14.05.1963.

17 AMPG, II. Abt., Rep. 60, Nr. 52.SP. Niederschrift der Sitzung des Senats der MPG am 14.12.1965.

18 The traditions of corporatist organization of politics, economy and society in Germany reach back to the German Empire. Research on the German variant of corporatism has so far mostly focused on the fields of economic, health or labor market policy, while the corporatist design of research policy can still largely be considered a desideratum (Abelshauser 1984: 311–313; Weßels 1999: 87–92).

19 In the early 1960s, the presidents of the Alliance of Science Organisations also met with the Federal Ministry of Defence or with the Minister-Presidents of the German states for talks. See for example AMPG, II. Abt., Rep. 69, Nr. 334. Auszug aus der Niederschrift der Sitzung des Wissenschaftlichen Rates der MPG am 14.05.1963; AMPG, III. Abt. Rep. 84–2, Nr. 6812. Aktenvermerk über eine Besprechung beim BMVtdg am 05.06.1962.

20 AMPG, II. Abt., Rep. 57, Nr. 334. Auszug aus der Niederschrift der Sitzung des Verwaltungsrates der MPG am 14.02.1966.

21 BArch B 196/16341. Interner Vermerk des BMFT bzgl. der Einladung des Präsidentenkreises vom 01.02.1974.

22 Ibid. Entwurf eines Einladungsschreibens für die Sitzung des Präsidentenkreises am 12.03.1974.

23 BArch, B 136/33238. Interner Vermerk des BMFT bzgl. der Sitzung des Präsidentenkreises am 17.04.1978.

24 Hans Matthöfer was the only one during this period who stayed in office for at least four years before becoming Minister of Finance in 1978.

25 BArch, B 196/19557. Interner Vermerk des BMFT zur Vorbereitung des Präsidentenkreises am 18.12.1980.

26 BArch, B 196/51378. Interner Vermerk des BMFT bzgl. der Sitzung des Präsidentenkreises am 16.11.1982.

27 BArch, B 136/33238. Interner Vermerk des BMFT bzgl. der Sitzung des Präsidentenkreises am 16.11.1982

28 See for example DFGA, AZ 02219-04, Bd. 1a. Vermerk der DFG über die Sitzung der Heiligen Allianz am 26.02.1974; Ibid. Vermerk der DFG über die Sitzung der “Heiligen Allianz” am 27.11.1974; DFGA, AZ 02219-04, Bd. 2. Vermerk der DFG über die Sitzung der “Heiligen Allianz” am 02.09.1975.

29 For an overview of the topics discussed, see the invitation letters and minutes of the respective meetings during the 1970s and 1980s in BArch, B 196. Questions of funding and financial planning were discussed by the ministry and the Alliance quite regularly, see e.g. BArch, B 196/16343. Vermerk des BMFT über die Sitzung des Präsidentenkreises am 05.09.1975, BArch, B 196/19554. Vermerk des BMFT über die Sitzung des Präsidentenkreises am 31.10.1979.

30 See e.g. BArch, B 196/19557. Vermerk des BMFT über die Sitzung des Präsidentenkreises am 18.12.1980, BArch, B 196/103437 Vermerk des BMFT über die Sitzung des Präsidentenkreises am 23.02.1987.

31 These included the Nuclear Research Center Karlsruhe (KfK), the Nuclear Research Facility Jülich (KFA), the *Gesellschaft für Kernenergieverwertung in Schiffbau und Schifffahrt* (GKSS), the *Hahn-Meitner-Institut* (HMI), the German Electron Syncrotron (DESY), the Max Planck Institute of Plasma Physics (IPP), the German Society for Radiation Research (GSF), the German Test and Research Institute for Aviation and Space Flight (DFVLR), the Society for Mathematics and Information technology (GMD) und the German Society for Heavy Iron Research (GSI).

32 DFGA, AZ 02219-04, Bd. 2. Vermerk der DFG über die Sitzung der Allianz am 26.04.1976.

33 At the same time, the FhG was also newly included in the Presidential Circle. However, it took until 1981 until the FhG was also invited to the meetings of the Alliance.

34 See for example DFGA, AZ 02219-04, Bd. 2. Vermerk der DFG über die Sitzung der Allianz am 05.11.1975.

35 Ibid. Vermerk der DFG über die Sitzung der Allianz am 26.04.1976.

36 Ibid.

37 AGF‑G, 6.4.3. Heisenberg Programm, Auszug aus dem Protokoll der AGF Mitgliederversammlung am 19./20.10.1977. Cited according to Szöllösi-Janze (1990b: 308).

38 BArch, B 388/2. Interner Vermerk zu den Highlights der Amtszeit von Prof. Schopper vom 28.02.1989.

39 Ibid. Interner Vermerk zu den Highlights der Amtszeit von Prof. Schopper vom 28.02.1989.

40 BArch, B 388/1. Gesprächsnotiz von G. zu Putlitz über ein Treffen mit dem Bundespräsidenten am 12.01.1981.

41 Ibid. Aktennotiz von G. zu Putlitz über die Sitzung der Allianz am 04.03.1981.

42 Ibid. Gedächtnisprotokoll von G. zu Putlitz über die Sitzung des Präsidentenkreises am 11.06.1981.

43 Ibid. Aktennotiz von G. zu Putlitz zu einem Gespräch mit Reimar Lüst und Dietrich Ranft (MPG) am 21.01.1982.

44 Ibid.

45 Ibid.

46 In total, only the DFG chaired three more meetings of the Alliance than the WR. One reason for this is that in 2000 the Alliance decided to rotate the chairmanship for their meetings on an annual basis instead of from one meeting to the next. The DFG was the first science organization to take over the annual chairmanship in April 2000 and thus chaired the two remaining meetings in this year.

47 AMPG, II. Abt., Rep. 57, Nr. 607, Bd. 2. Schreiben von FhG, MPG und AGF an H. Riesenhuber (BMFT) vom 21.06.1985.

48 AMPG, II. Abt., Rep. 57, Nr. 1398. Interner Vermerk der MPG über die Sitzung der Allianz am 04.11.1992; AMPG, II. Abt., Rep. 57, Nr. 613, Bd. 1. Interne Notiz der MPG zum Thema Verbundforschung für den Generalsekretär vom 05.01.1993.

49 See for example AMPG, II. Abt., Rep. 57, Nr. 612, Bd. 1. Interner Vermerk der MPG über die Sitzung der Allianz vom 24.06.1992. In autumn 1992, the Alliance addressed a memorandum and an accompanying letter to the Minister-Presidents of the Federal States, various Federal Ministers and the chairmen of corresponding committees in the Bundestag in order to prevent a tightening of the guidelines for animal experiments in the course of the amendment of the Animal Protection Act.

## References

[CR1] Abelshauser W (1984). The First Post-liberal Nation: Stages in the Development of Modern Corporatism in German. European History Quarterly.

[CR2] Alter P (2000). Der DAAD in der Zeit: Geschichte, Gegenwart und zukünftige Aufgaben; vierzehn Essays.

[CR3] Ash MG, vom Bruch R, Kaderas B (2002). Wissenschaft und Politik als Ressourcen füreinander. Wissenschaften und Wissenschaftspolitik: Bestandsaufnahmen zu Formationen, Brüchen und Kontinuitäten im Deutschland des 20. Jahrhunderts.

[CR4] Ash MG (2010). Wissenschaft und Politik: Eine Beziehungsgeschichte im 20. Jahrhundert. Archiv für Sozialgeschichte.

[CR5] Ash MG, Flachowsky S, Hachtmann R, Schmaltz F (2016). Reflexionen zum Ressourcenansatz. Ressourcenmobilisierung: Wissenschaftspolitik und Forschungspraxis im NS-Herrschaftssystem.

[CR6] Bartz O (2007). Der Wissenschaftsrat: Entwicklungslinien der Wissenschaftspolitik in der Bundesrepublik Deutschland 1957–2007.

[CR50] van Bebber F (2011). Ritterrunde im Verborgenen. duz.

[CR7] Bogner A, Falk S, Rehfeld D, Römmele A, Thunert M (2006). Politikberatung im Politikfeld der Biopolitik. Handbuch Politikberatung.

[CR8] Bourdieu P (1985). Sozialer Raum und “Klassen”.

[CR9] Bourdieu P (1992). Die verborgenen Mechanismen der Macht.

[CR10] Brankovic J, Ringel L, Werron T (2018). How rankings produce competition: The case of global university rankings. Zeitschrift für Soziologie.

[CR11] Braun D (1997). Die politische Steuerung der Wissenschaft.

[CR12] Brill A (2017). Von der „Blauen Liste“ zur gesamtdeutschen Wissenschaftsorganisation: Die Geschichte der Leibniz Gemeinschaft.

[CR13] Fehrenbach E (2008). Vom Ancien Régime zum Wiener Kongreß.

[CR14] Fisch S, Rudloff W (2004). Experten und Politik: Wissenschaftliche Politikberatung in geschichtlicher Perspektive.

[CR15] Frevert U (2013). Vertrauensfragen: Eine Obsession der Moderne.

[CR16] Gerstengarbe S, Thiel J, vom Bruch R (2016). Die Leopoldina: Die Deutsche Akademie der Naturforscher zwischen Kaiserreich und früher DDR.

[CR17] Goffman E (1969). Wir alle spielen Theater: Die Selbstdarstellung im Alltag.

[CR18] Hochschulrektorenkonferenz 2020. Alliance of Science Organisations in Germany. https://www.hrk.de/hrk-at-a-glance/alliance-of-science-organisations-in-germany/. Accessed 16 December 2021.

[CR19] Hoffmann D, Trischler H, Mlynek J, Bittner A (2015). Die Helmholtz-Gemeinschaft in historischer Perspektive. 20 Jahre Helmholtz-Gemeinschaft.

[CR20] Hohn H-W, Simon D, Knie A, Hornborstel S, Zimmermann K (2016). Governance-Strukturen und institutioneller Wandel des außeruniversitären Forschungssystems Deutschlands. Handbuch Wissenschaftspolitik.

[CR21] Hohn H-W, Schimank U (1990). Konflikte und Gleichgewichte im Forschungssystem: Akteurskonstellationen und Entwicklungspfade der staatlich finanzierten außeruniversitären Forschung.

[CR22] Klofat R (1991). Herrenhaus der Wissenschaft.

[CR23] Luhmann N (2014). Vertrauen: ein Mechanismus der Reduktion sozialer Komplexität.

[CR24] May T, Vereinigung der Kanzlerinnen und Kanzler der Universitäten Deutschlands (2018). Differenzierungsprozesse fördern, gestalten und stabilisieren: Die Bedeutung von Kooperation und Konkurrenz für die Leistungsfähigkeit des Wissenschaftssystems. Kooperation und Konkurrenz: Universitäten und ihre Partner unter verschärften Wettbewerbsbedingungen?.

[CR25] Mayntz R, Scharpf FW (1995). Gesellschaftliche Selbstregelung und politische Steuerung.

[CR26] Nickelsen K, Jessen R (2014). Kooperation und Konkurrenz in den Naturwissenschaften. Konkurrenz in der Geschichte: Praktiken – Werte – Institutionalisierungen.

[CR27] Nickelsen K, Krämer F (2016). Introduction: Cooperation and Competition in the Sciences. NTM Zeitschrift für Geschichte der Wissenschaften Technik und Medizin.

[CR28] Orth K (2011). Autonomie und Planung der Forschung: Förderpolitische Strategien der Deutschen Forschungsgemeinschaft 1949–1968.

[CR29] Patzwaldt K, Buchholz K, Falk S, Rehfeld D, Römmele A, Thunert M (2006). Politikberatung in der Forschungs- und Technologiepolitik. Handbuch Politikberatung.

[CR30] Pyta W, Kroll F-L (1996). Idee und Wirklichkeit der “Heiligen Allianz”. Neue Wege der Ideengeschichte: Festschrift für Paul Kluxen zum 85. Geburtstag.

[CR31] Ritter GA, Szöllösi-Janze M, Trischler H (1999). Antworten auf die amerikanische Herausforderung: Forschung in der Bundesrepublik und der DDR in den “langen” siebziger Jahren.

[CR32] Röhl HC (1994). Der Wissenschaftsrat: Kooperation zwischen Wissenschaft, Bund und Ländern und ihre rechtlichen Determinanten.

[CR33] Rudloff W, Fisch S, Rudloff W (2004). Einleitung: Politikberatung als Gegenstand historischer Betrachtung: Forschungsstand, neue Befunde, übergreifende Fragestellungen. Experten und Politik: Wissenschaftliche Politikberatung in geschichtlicher Perspektive.

[CR34] Rudloff W (2005). Does Science Matter? Zur Bedeutung wissenschaftlichen Wissens im politischen Prozess am Beispiel der bundesdeutschen Bildungspolitik in den Jahren des “Bildungsbooms”.

[CR35] Rusinek BA (1996). Das Forschungszentrum: Eine Geschichte der KFA Jülich von ihrer Gründung bis 1980.

[CR36] Schimank U, Mayntz R, Scharpf FW (1995). Politische Steuerung und Selbstregulation des Systems organisierter Forschung. Gesellschaftliche Selbstregelung und politische Steuerung.

[CR37] Schimank U (2009). Planung – Steuerung – Governance: Metamorphosen politischer Gesellschaftsgestaltung. Die Deutsche Schule: Zeitschrift für Erziehungswissenschaft, Bildungspolitik und pädagogische Praxis.

[CR38] Seefried E (2010). Experten für die Planung? Zukunftsforscher als Berater der Bundesregierung 1966–1972/73. Archiv für Sozialgeschichte.

[CR39] Simmel G, Dahme H-J, Rammstedt O (1986). Soziologie der Konkurrenz. Schriften zur Soziologie: Eine Auswahl.

[CR40] Soutschek L, Nickelsen K (2019). „Zusammenwirken“ oder „Wettstreit der Nationen“. NTM Zeitschrift für Geschichte der Wissenschaften Technik und Medizin.

[CR41] Stamm T (1981). Zwischen Staat und Selbstverwaltung: Die deutsche Forschung im Wiederaufbau 1945–1965.

[CR42] Stucke A (1993). Institutionalisierung der Forschungspolitik.

[CR43] Stucke A, Falk S, Rehfeld D, Römmele A, Thunert M (2006). Der Wissenschaftsrat. Handbuch Politikberatung.

[CR44] Stucke A, Simon D, Knie A, Hornborstel S, Zimmermann K (2016). Staatliche Akteure der Wissenschaftspolitik. Handbuch Wissenschaftspolitik.

[CR45] Szöllösi-Janze M, Szöllösi-Janze M, Trischler H (1990). Die Arbeitsgemeinschaft der Großforschungseinrichtungen: Identitätsfindung und Selbstorganisation. 1958–1970. Großforschung in Deutschland.

[CR46] Szöllösi-Janze M (1990). Geschichte der Arbeitsgemeinschaft der Großforschungseinrichtungen: 1958–1980.

[CR47] Szöllösi-Janze M, Trischler H (1990). Großforschung in Deutschland.

[CR48] Trischler H, Szöllösi-Janze M, Trischler H (1990). Planungseuphorie und Forschungssteuerung in den 1960er Jahren in der Luft- und Raumfahrtforschung. Großforschung in Deutschland.

[CR49] Trischler H, vom Bruch R (1999). Forschung für den Markt: Geschichte der Fraunhofer-Gesellschaft.

[CR51] Vogt M, Kirchhoff T (2015). Konkurrenz und Solidarität: Alternative oder verwobene Formen sozialer Interkation. Konkurrenz: Historische, strukturelle und normative Perspektiven.

[CR52] Walker M, Orth K, vom Bruch R, Ulrich H (2013). The German Research Foundation 1920–1970.

[CR53] Weingart P (1983). Verwissenschaftlichung der Gesellschaft – Politisierung der Wissenschaft. Zeitschrift für Soziologie.

[CR54] Weingart P (2005). Die Stunde der Wahrheit?.

[CR55] Wenninger A, Will F, Dickel S, Maasen S, Trischler H, Zachmann K, Ehlers S (2019). Ein- und Ausschließen: Evidenzpraktiken in der Anthropozändebatte und der Citizen Science. Wissen und Begründen: Evidenz als umkämpfte Ressource in der Wissensgesellschaft.

[CR56] Werron T, Tyrell H, Rammstedt O, Meyer O (2011). Zur sozialen Konstruktion moderner Konkurrenzen. Das Publikum in der “Soziologie der Konkurrenz”. Georg Simmels große “Soziologie”: Eine kritische Sichtung nach hundert Jahren.

[CR57] Weßels B, Kaase M, Schmid G (1999). Die deutsche Variante des Korporatismus. Eine lernende Demokratie: 50 Jahre Bundesrepublik Deutschland.

[CR58] Zierold K (1968). Forschungsförderung in drei Epochen: Deutsche Forschungsgemeinschaft; Geschichte, Arbeitsweise, Kommentar.

